# Comparative analysis of within-host diversity among vaccinated COVID-19 patients infected with different SARS-CoV-2 variants

**DOI:** 10.1016/j.isci.2022.105438

**Published:** 2022-10-25

**Authors:** Hebah A. Al-Khatib, Maria K. Smatti, Fatma H. Ali, Hadeel T. Zedan, Swapna Thomas, Muna N. Ahmed, Reham A. El-kahlout, Mashael A. Al Bader, Dina Elgakhlab, Peter V. Coyle, Laith J. Abu-Raddad, Asma A. Al Thani, Hadi M. Yassine

**Affiliations:** 1 Biomedical Research Center, Qatar University, Doha 2713, Qatar; 2 Virology Laboratory, Hamad Medical Corporation, Doha 3050, Qatar; 3 National Reference Laboratory, Ministry of Public Health, Doha 42, Qatar; 4 Qatar Biobank, Qatar Foundation, Doha, Qatar; 5 Weill Cornell Medicine–Qatar, Qatar Foundation, Doha, Qatar; 6 Department of Biomedical Sciences, College of Health Sciences, Qatar University, Doha, Qatar

**Keywords:** Immunology, Immune response, Virology, Genomics

## Abstract

Severe acute respiratory syndrome coronavirus 2 (SARS-CoV-2) is a rapidly evolving RNA virus that mutates within hosts and exists as viral quasispecies. Here, we evaluated the within-host diversity among vaccinated and unvaccinated individuals (n = 379) infected with different SARS-CoV-2 Variants of Concern. The majority of samples harbored less than 14 intra-host single-nucleotide variants (iSNVs). A deep analysis revealed a significantly higher intra-host diversity in Omicron samples than in other variants (p value < 0.05). Vaccination status and type had a limited impact on intra-host diversity except for Beta-B.1.315 and Delta-B.1.617.2 vaccinees, who exhibited higher diversity than unvaccinated individuals (p values: <0.0001 and <0.0021, respectively). Three immune-escape mutations were identified: S255F in Delta and R346K and T376A in Omicron-B.1.1.529. The latter 2 mutations were fixed in BA.1 and BA.2 genomes, respectively. Overall, the relatively higher intra-host diversity among vaccinated individuals and the detection of immune-escape mutations, despite being rare, suggest a potential vaccine-induced immune pressure in vaccinated individuals.

## Highlights

• Higher within-host diversity among omicron-positive samples
• Higher within-host diversity among vaccinated individuals regardless of virus lineage
• Limited impact of vaccine type on within-host diversity of SARS-CoV-2

## Graphical abstract

**Figure:**
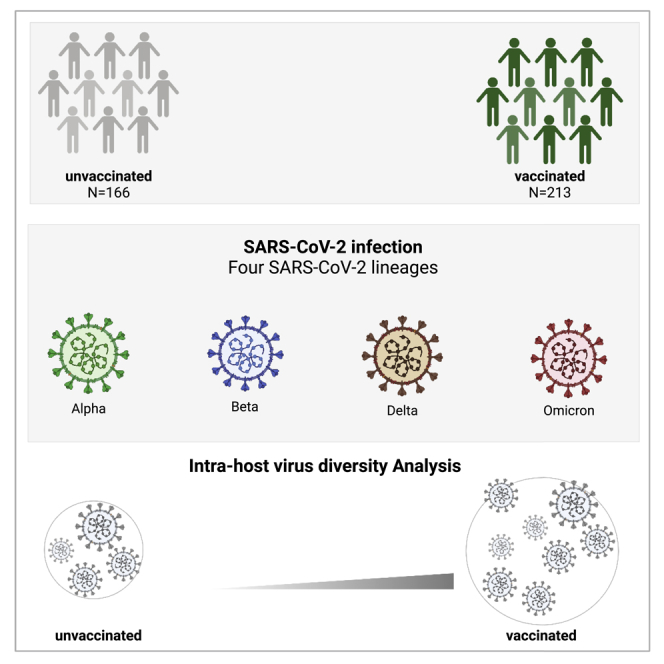

## Introduction

Since its emergence in November 2019, severe acute respiratory syndrome coronavirus 2 (SARS-CoV-2) has evolved rapidly, accumulating mutations and generating new variants.^1^^,^^2^^,^^3^^,^^4^ Emerging variants show variable characteristics of transmissibility, virulence, and immune evasion.^5^^,^^6^^,^^7^ Based on these characteristics, the Centers for Disease Control and Prevention (CDC) has classified the new variants into “variants of concern,” “variants of interest,” and “variants under monitoring.”^8^^,^^9^ Recent variants of concern include the Delta and Omicron variants, which have caused the third and fourth waves of infection in many countries, respectively. The 2 variants are characterized by increased transmissibility and reduced neutralization by postvaccination sera.^6^^,^^10^^,^^11^^,^^12^ As SARS-CoV-2 continues to circulate globally, several genetic mutations have accumulated and will continue to accumulate, possibly at a faster rate as greater immunity develops in the population. The origin of SARS-CoV-2 variants remains unclear, and there is no clear evidence explaining the mechanism(s) that led to their emergence. Several hypotheses have been proposed including (i) virus evolution in animals (zoonotic origin), (ii) virus evolution in long-term infected immunocompromised individuals, and (iii) virus evolution in immunocompetent individuals with pre-existing immunity due to vaccination, infection, treatment with convalescent sera, and monoclonal antibodies.

The evolution of coronaviruses, like other RNA viruses, begins with the accumulation of mutations as the virus replicates within hosts. Therefore, coronaviruses exist within hosts as a cloud of genomes referred to as within-host diversity (quasispecies). Of within host mutations, only few may rise in frequency, transmit to other hosts, or even fix in the virus population.^13^^,^^14^ Factors that determine within-host evolution of RNA viruses are not well understood. Multiple factors may affect within-host evolution of RNA viruses including antigenic selection, antiviral treatment, tissue specificity, spatial structure, and multiplicity of infection.^15^^,^^16^^,^^17^ Potentially advantageous mutations that confer enhanced receptor binding affinity, increased transmissibility, and immune-escape properties might be selected and become dominant.^18^^,^^19^ Recently, concerns have been raised that expanding massive vaccination could increase within-host selection for vaccine-escape mutations, ultimately undermining vaccine effectiveness.^20^^,^^21^ Within-host SARS-CoV-2 diversity was commonly reported in COVID-19 patients, particularly among immunocompromised patients with persistent infection.^22^^,^^23^^,^^24^^,^^25^ Investigating the within-host evolution of SARS-CoV-2 in immunocompromised patients revealed a dynamic within-host diversity that continues to change throughout the course of the infection.^23^^,^^24^ More importantly, the lack of an effective immune response in those patients allowed for a relaxed within-host virus evolution which resulted in the emergence of immune-escape mutations, many of which were found in other variants of concern (Alpha and Beta).^23^

Published data in immunocompetent patients reported variable levels of within-host diversity among COVID-19 patients.^22^^,^^25^^,^^26^ These differences could be attributed to host- and virus-related factors such as age, underlying comorbidities, and SARS-CoV-2 lineage. We have previously shown that higher within-host diversity is commonly seen among elderly patients (>60 years old) and patients with severe respiratory symptoms.^22^ Here, we evaluated within-host diversity among non-hospitalized symptomatic COVID-19 patients infected with different SARS-CoV-2 variants of concern. We sequenced 379 SARS-CoV-2 genomes from samples collected during the 4 waves of infection in Qatar. The 4 waves were caused by Alpha, Beta, Delta, and Omicron variants, respectively. Samples were subdivided based on vaccination status and vaccination type to compare within-host diversity between vaccinated and unvaccinated individuals and to investigate the possible emergence of immune-escape mutations among vaccinated individuals.

## Results

### Evaluating within-host diversity of SARS-CoV-2

**Figure 1:**
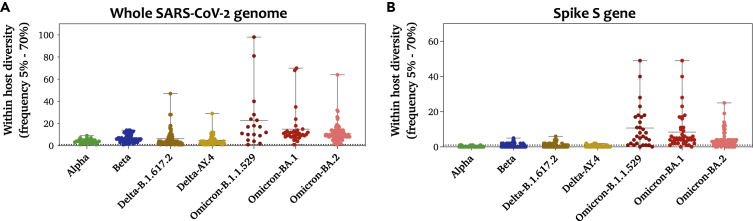
Number of iSNVs (MAF > 0.05) observed in each sample of the SARS-CoV-2 lineages/sub-lineages (A) Number of iSNVs seen in the whole genome of each sample. (B) Number of iSNVs seen in the spike gene of each sample. All mutations were called with respect to the Wuhan Hu-1 reference sequence (GenBank: NC_045512). Data represent the mean of the mean number of iSNVs ± SD reported among samples that belong to the same lineage/sub-lineage. All samples (n = 379) in our dataset are included in this figure. Number of samples within each group is listed in Table 1.

**Figure 2:**
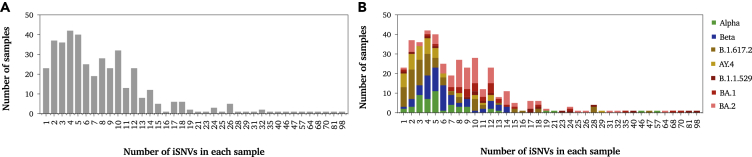
Histograms showing the number of samples exhibiting N number of iSNVs (MAF > 0.05) (A) Histogram showing the total number of samples with N number of iSNVs. (B) Stacked histogram showing the number of samples that had N number of iSNVs sub-categorized based on lineage: Alpha, Beta, Delta, and Omicron lineages and sub-lineages. All identified sites were included in this figure except for those located in intragenic regions and in the upper and lower untranslated regions of the genome.

To evaluate the within-host diversity of SARS-CoV-2, we called all intra-host single-nucleotide variants (iSNVs) occurring above an MAF of 0.05 in each of the analyzed samples. A total of 379 samples were included in the analysis, of which 213 were vaccinated with either of mRNA vaccines: mRNA-1273 (N=101) or BNT162b2 (N=112) (Table 1). Overall, low levels of within-host diversity (less than 14 iSNVs) were reported among the majority of samples regardless of SARS-CoV-2 lineage (Figure 1A). As expected, within-host diversity was significantly higher in Omicron-positive samples than that in other lineages (Table 2). On average, Omicron-positive samples exhibited the highest number of iSNVs (mean = 14, SD = 11.3), followed by Delta-B.1.1617.2 (mean = 6, SD = 7.5) and Beta (mean = 6, SD = 3.4), while the lowest diversity was reported among Alpha- and Delta-AY.4-positive samples (mean = 4). In all lineages, the total number of mutations in the virus genome was proportional to the number of mutations in the spike (S) gene (Figure 1B). All samples harbored at least 1 iSNV (Figure 2A). The majority of samples had less than 14 iSNVs regardless of lineage. Eight samples had a higher number of iSNVs ranging from 30 to 70 iSNVs; however, the higher diversity within those samples was not associated with a particular lineage (Figure 2B). Those samples belonged to Alpha, Delta-B.1.617.2, BA.1, and BA.2 lineages.

**Table 1:** Numbers of SARS-CoV-2 sequences within each group/subgroup included in this study

Lineage	Sub-lineage	Vaccinated		Unvaccinated	Total	Sample collection time period
		mRNA-1273	BNT162b2			
Alpha		–	–	33	33	December-January, 2021
Beta		12	29	33	74	March-May, 2021
Delta	B.1.617.2	22	17	30	69	July-August, 2021
	AY.4	12	15	22	49	September-October, 2021
Omicron	B.1.1.529	8	9	4	21	December, 2021
	BA.1	12	10	10	32	December 2021-January 2022
	BA.2	35	32	34	101	December 2021-January 2022

**Table 2:** Comparison of within-host diversity between omicrons and other SARS-CoV-2 lineages

Multiple comparisons test	Adjusted p value	Summary
Alpha vs. Omicron-B.1.1.529	0.0024	∗
Alpha vs. Omicron-BA.1	<0.0001	∗∗∗∗
Alpha vs. Omicron-BA.2	<0.0001	∗∗∗∗
Beta vs. Omicron-B.1.1.529	0.4078	ns
Beta vs. Omicron-BA.1	0.0059	∗
Beta vs. Omicron-BA.2	0.0095	∗
Delta-B.1.617.2 vs. Omicron-B.1.1.529	0.0039	∗
Delta-B.1.617.2 vs. Omicron-BA.1	<0.0001	∗∗∗∗
Delta-B.1.617.2 vs. Omicron-BA.2	<0.0001	∗∗∗∗
Delta-AY.4 vs. Omicron-B.1.1.529	0.0010	∗∗∗
Delta-AY.4 vs. Omicron-BA.1	<0.0001	∗∗∗∗
Delta-AY.4 vs. Omicron-BA.2	<0.0001	∗∗∗∗

Significance is indicated as follows: ∗ for <0.03, ∗∗ for <0.0021, ∗∗∗ for <0.0002, and ∗∗∗∗ for <0.0001.

**Figure 3:**
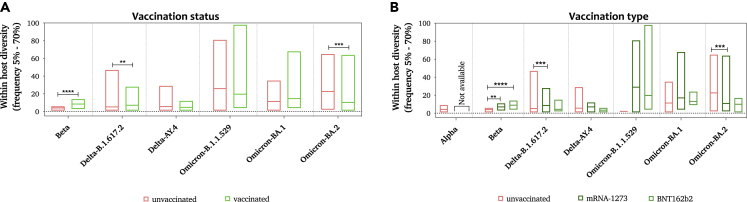
Average number of iSNVs observed in SARS-CoV-2-positive samples Number of iSNVs seen in each sample of the SARS-CoV-2 lineages sub-divided based on (A) vaccination status (vaccinated and unvaccinated) and (B) mRNA vaccine type (mRNA-1273 [Moderna] and BNT162b2 [Pfizer]). All mutations were included except for those located in intragenic regions and in the upper and lower untranslated regions of the genome. Each bar represents the mean iSNVs value ±SD. Significance is indicated as follows: ∗ for <0.033, ∗∗ for <0.0021, ∗∗∗ for <0.0002, and ∗∗∗∗ for <0.0001. A detailed linear model analysis that incorporates the interaction between the virus lineage and vaccine type is demonstrated in Figure S1 and File S1.

Then, we evaluated the impact of the vaccine on within-host diversity. While vaccination status did not seem to affect the within-host diversity in Omicron-positive samples, significant differences were seen between vaccinated and unvaccinated samples collected from Beta-positive (p value < 0.001) and Delta-B.1.617.2-positive (p value < 0.001) samples. Intriguingly, this significance was driven by Pfizer-vaccinated individuals in Beta-positive samples (p value < 0.001) and by Moderna-vaccinated individuals in Delta-B.1.617.2-positive samples (Figure 3). Lower within-host diversity was reported among BA.1 and BA.2 individuals who received three doses of the vaccine compared to those who received two doses. Moderna-vaccinated individuals who received their third dose have generally exhibited lower diversity than those who received two doses. However, only a few samples were collected from individuals who received three doses of the vaccine, so no confirmative conclusions could be drawn from this finding. We have also investigated the correlation between within-host diversity and the duration between vaccination and infection; however, no correlation was seen regardless of lineage, vaccination status, or vaccination type. A linear model was also performed to study the interaction effect of lineage and vaccine type on the prevalence of iSNVs. An analysis of the interaction effect showed no significance effect of vaccine type on iSNVs regardless of SARS-CoV-2 lineage (Figure S1).

### Distribution of iSNVs across the genome

**Figure 4:**
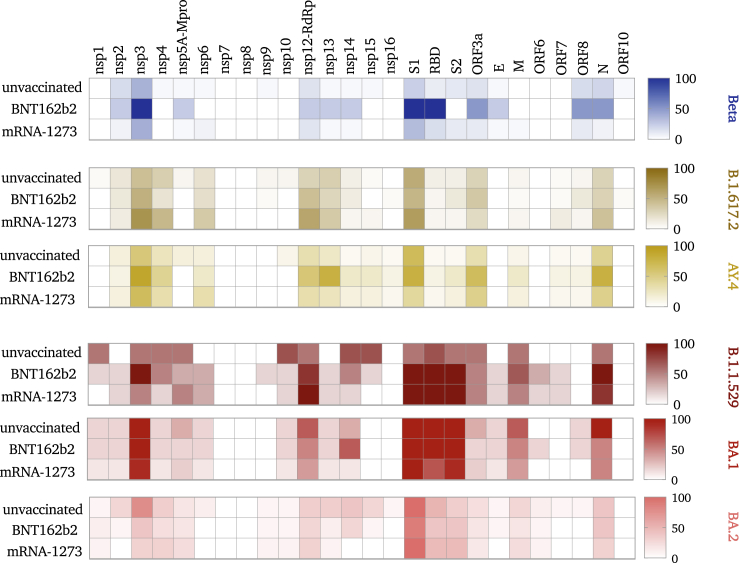
Heatmap demonstrating the distribution of iSNVs throughout SARS-CoV-2 genome of each lineage All identified intra-host variation sites (MAF > 0.05) were included in the heatmap analysis except for those located in intragenic regions and the upper and lower untranslated regions of the genome. The range on the right demonstrates the number of intra-host variations sites in each region of the genome where 0 indicates no mutations while 100 indicates that 100 mutations were found in this region.

We next looked at the distribution of the identified iSNV sites across the genome. In all groups, the majority of iSNVs were found in the 3′ and 5′ untranslated (UTRs) and intragenic regions of SARS-CoV-2 sequences, and those were excluded from subsequent analyses. Overall, lineages exhibited variable numbers of iSNV sites: 40 iSNVs in Alpha sequences, 59 in Omicron-B.1.1.529, 57 in Omicron-BA.1, 61 in Omicron-BA.2, 77 in Delta-AY.4 sequences, and 79 in Beta sequences. The largest number of iSNV sites, however, was reported in Delta-B.1.617.2 sequences (n = 188 iSNVs). The majority of iSNV sites in Omicron sequences are lineage-specific mutations that are mainly found in the S gene. On the other hand, the majority of mutations in Beta and Delta sequences are non-lineage-specific mutations that are distributed across the genome. In all lineages, the distribution of iSNVs across the genes was considerably variable, with open-reading frames (ORFs) ORF1ab, 3a, nucleocapsid (N), and spike (S) genes showing the highest densities (Figure 4). The majority of mutations in ORF1ab were localized in nsp3 and nsp12 (RNA-dependent RNA polymerase [RdRp]). The higher number of iSNVs in these two regions was associated with higher iSNV numbers in the receptor-binding domain (RBD) of S1, ORF3a, ORF8, and N genes. This was particularly seen in sequences from mRNA-1273-vaccinated individuals infected with Beta or Delta-AY.4 and from BNT162b2-vaccinated individuals infected with Delta-B.1.617.2 (Figure 4).

### In-depth analysis of non-synonymous, low-frequency mutations

Among the identified within-host mutations, only a few may rise in frequency and possibly transmit to other hosts.^15^ Here, we focused the analysis on non-synonymous mutations with the MAF range of 0.05–0.5 and evaluated their emergence, frequency, and prevalence among vaccinated and unvaccinated individuals. Only iSNV sites not detected in the control and found in more than 2% of samples were included in the subsequent analysis.

**Figure 5:**
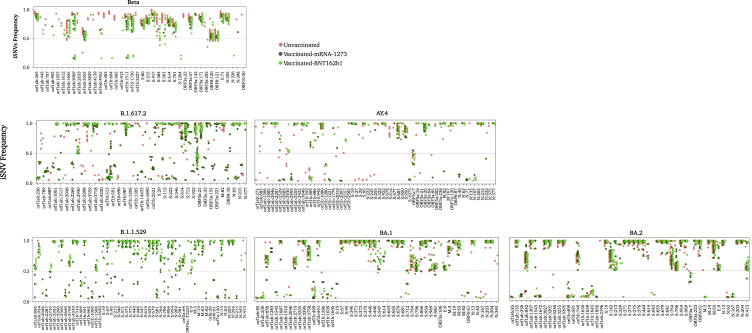
Distribution and frequency of the non-synonymous mutations (MAF > 0.05) across SARS-CoV-2 genomes of each lineage

**Figure 6:**
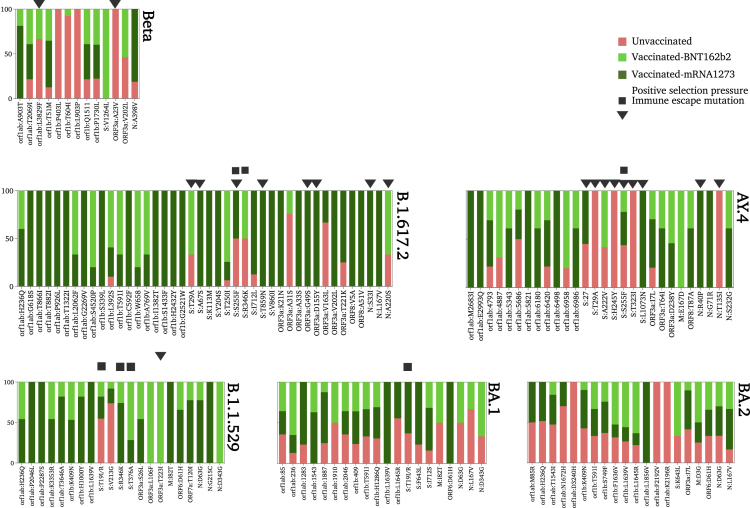
Prevalence of non-lineage mutations identified in vaccinated and unvaccinated individuals of each lineage This figure displays non-synonymous mutations that showed significant differences in their prevalence among the three groups: unvaccinated, Moderna-vaccinated, and Pfizer-vaccinated individuals. The squares and triangles above the bars indicate immune-escape mutations and positively selected site, respectively.

In Alpha and Beta sequences, the vast majority of identified iSNVs were high-frequency, lineage-specific mutations (Figure 5). In Beta sequences, high-frequency, non-lineage mutations were found in ORF1ab (n = 9), ORF3a (n = 2), and N (n = 1). Two mutations, L3829F in ORF1ab and A23V in ORF3a, were found to be under positive selection. Only one low-frequency, non-lineage mutation was found, S: V1264L, and it was found in 5% of Beta samples vaccinated with BNT162b2 (Figure 6). Some mutations showed variable frequencies among the groups. The V202L mutation in ORF3a, for example, was found at low frequency (<0.5) in BNT162b2-vaccinated individuals and at higher frequencies (>0.5) in unvaccinated individuals. Notably, none of the mutations in Beta sequences were associated with immune escape (Figure 6).

Unlike Alpha and Beta sequences, Delta-B.1.617.2 exhibited a high number (n = 42) of both high- and low-frequency non-lineage mutations, particularly in the S gene (n = 10 iSNVs) (Figure 5). Four mutations (out of 10 in S) were under positive selection: T29A, A67S, S255F, and T859N (Figure 6). The S255F mutation in the N-terminal domain (NTD) of the S gene is also an escape mutant that demonstrated reduced neutralization by the potent NTD monoclonal antibody, mAb_S2L28.^27^ Another immune-escape mutation that was found in the RBD of S protein is R346K. This mutation showed resistance to monoclonal antibodies such as C135. Of note, this mutation was detected later on at a higher prevalence in the Omicron variant B.1.1.529 and was fixed in all BA.1 sequences (Figure 6).

Delta-B.1.617.2 sequences have also exhibited a high number of ORF1ab mutations (n = 19), seven of which are in the RdRp coding region, the highest compared to other lineages. Interestingly, six (out of seven) of RdRp mutations were found only in vaccinated individuals. This could partially explain the higher number of iSNVs in Delta-B.1.617.2 samples collected from mRNA-1273-vaccinated individuals.

An analysis of low-frequency mutations in Delta-B.1.617.2, in particular, revealed a large number of low-frequency mutations (n = 33) compared to other lineages. Low-frequency mutations were found in ORF1ab (n = 18), S (n = 8), ORF3a (n = 3), ORF8 (n = 2), and N (n = 2). Notably, the majority (24 out of 33) of identified low-frequency mutations were detected in vaccinated samples, particularly in mRNA-1273-vaccinated individuals. The prevalence of low-frequency mutations among mRNA-1273-vaccinated individuals was variable ranging from 9% to 18% (Figure 6). While this may suggest a possible transmission of these low-frequency mutations, their emergence in mRNA-vaccinated individuals exclusively may favor the *de novo* emergence assumption rather than transmission. Seven of these mutations were found to be under positive selection pressure but not associated with immune escape: four in the S gene, two in ORF 3a, and 2 in the N gene (Figure 6).

The other Delta variant, AY.4, has also exhibited a relatively high number of non-lineage-specific mutations (n = 28). Unlike Delta-B.1.617.2, low-frequency mutations were less common among Delta-AY.4 samples. Mutations were located in ORF1ab (n = 12), S (n = 7), ORF3a (n = 3), and N (n = 4). All non-lineage mutations in S were found at high frequency, except for K1073N which was detected at low frequency (<0.1) in 33% of mRNA-1273-vaccinated individuals. The rest of the non-lineage mutations, on the other hand, did not show any specific association with vaccination status and/or type. Moreover, six mutations (out of seven) in the S gene were found to be under positive selection pressure. Two S mutations, S255F and T29A, were also found in Delta-B.1.617.2 sequences; however, S255F prevalence was higher in Delta-AY.4 samples regardless of vaccination status (Figure 6).

In ORF1ab, mutations were found in nsp3 (n = 1), nsp4 (n = 1), nsp13 (n = 9), and 3′-to-5′ exonuclease (nsp14A, n = 1) coding regions. Three of these mutations, M2683I, E2993Q, and K6498T, were found exclusively in mRNA-1273-vaccinated individuals (Figure 6). Only four (out of the 12 non-lineage mutations) were found at low frequency. The rest of the ORF1ab mutations were detected at the consensus sequence level of Delta-AY.4 sequences. The prevalence of three low-frequency mutations in ORF1b—A942V (RdRp), A1779V (exonuclease), and K2097T (exonuclease)—was higher in vaccinated samples.

Four non-lineage mutations were in the N gene; two were detected exclusively at low frequencies (<0.15) among vaccinated individuals: R40P and G71R. The other two were detected at consensus sequences of vaccinated (S232G) and unvaccinated (T135I) samples.

Omicron sequences generally exhibited a smaller number of non-lineage mutations. Each of the Omicron variants, B.1.1.529, BA.1, and BA.2, carried 22 non-lineage mutations. The majority of these mutations were located in ORF1ab, and fewer mutations were found in S, ORF6, and N genes (Figure 6). All Omicron variants shared five non-lineage mutations: ORF1ab: H236Q, ORF1b: L1639V, ORF6: D61H, N: D63G, and N: D343G. Interestingly, ORF1b: L1639V located in the exonuclease coding region (nsp14A2) was found at low frequency exclusively in 33% of Omicron-B.1.1.529 and BA.1 individuals vaccinated with the mRNA-1273 vaccine. The prevalence of this mutation was found later on—at low frequency—in more than 50% of BA.2 samples regardless of vaccination status. A similar pattern was also seen for D61H mutation in ORF6. It appeared first as a low-frequency mutation (<0.3) in 30% of Omicron-B.1.1.529 and BA.1 individuals who received mRNA-1273, then was seen in the consensus sequences of all BA.2 samples regardless of vaccination status, suggesting a possible selection of this mutation.

BA.1 and BA.2 shared additional six non-lineage mutations; all were low-frequency mutations. The only exceptions were ORF1ab mutation, T1543I, and ORF1b mutation, T591I, which appeared at high frequencies in BA.2 and BA.1 omicron sub-lineages, respectively.

Within the host non-lineage, spike mutations were limited among all the three Omicron variants (Figure 6). BA.1 and BA.2 shared 1 low-prevalent S mutation, S643L, which was found at the consensus sequence level in BA.1 samples and low frequency in BA.2. Two S mutations were identified as immune-escape mutations: R346K (in Omicron-B.1.1.529 and BA.1) and T376A (in Omicron-B.1.1.529). All these mutations were found at a low frequency in vaccinated individuals. Of these, R346K and T376A were found later on at high frequency and prevalence in the consensus sequences of BA.1 and BA.2, respectively.

## Discussion

SARS-CoV-2 has evolved rapidly since its emergence in 2019 and generated hundreds of variants that caused multiple waves of infection worldwide.^28^ Most analyses report on variants’ mutations observed in virus consensus genomes and neglect mutations that appear at the sub-consensus level which may affect the virus's characteristics.^15^^,^^19^ Here, we looked beneath the consensus to analyze the genetic variation within SARS-CoV-2 viral populations in individuals infected with four of the SARS-CoV-2 variants of concern. Overall, we reported low levels of within-host diversity among all samples regardless of causative SARS-CoV-2 variants. The limited number of within-host mutations can be attributed to the low mutation rate of coronaviruses (1.1 × 10^−3^ substitutions/site/year).^29^ Unlike other RNA viruses, coronaviruses exhibit a unique proofreading activity of its 3′-to-5′ exoribonuclease which may correct some of the errors that occur during replication.^30^ The limited number of within-host mutations in SARS-CoV-2 samples we reported here is consistent with other reported levels^24^^,^^31^^,^^32^ but lower than that in some other studies,^26^^,^^33^ likely reflecting differences in the immune status of participants, sample selection criteria, and variant calling methods. Higher within-host diversity is commonly reported among immunocompromised patients.^23^^,^^25^ The absence of immune pressure in immunocompromised patients allows the virus to replicate and accumulate mutations at a faster rate than viruses replicating in immunocompetent patients. Conversely, the infection in immunocompetent patients as the case in our study is usually a self-limiting infection with limited within-host diversity.^22^^,^^24^ The limited within-host diversity can also be attributed to the dynamics of SARS-CoV-2 infection. Studies have demonstrated a dynamic within-host diversity throughout infection in both immunocompetent and immunocompromised patients.^23^^,^^26^^,^^32^^,^^34^ A longitudinal study in an immunocompromised patient showed that virus diversity tends to increase during infection (after 14 days).^23^ In their study, Weigang and colleagues were able to identify several mutations, however, at later stages of infection in an immunocompromised patient.^23^ In this study, we analyzed patients’ samples collected within 1–3 days following symptoms onset, which may also explain the limited diversity and may not be reflecting the actual dynamicity of within-host diversity.

The emergence and diversity of within-host mutations in RNA viruses, including SARS-CoV-2, are driven by many factors including tissue specificity, antiviral treatment, and antigenic selection.^15^^,^^17^ Antigenic selection is one of the major contributors to within-host virus evolution. Immune pressure resulting from vaccination and/or infection could in theory maximize the within-host diversity and potentially speed up the virus evolution rate. Therefore, we investigated the impact of the vaccine on the within-host diversity of different lineages. As expected, vaccinated individuals exhibited an overall higher number of within-host mutations, particularly in Delta-B.1.617.2- and Beta-infected individuals. In Delta-B.1.617.2, higher diversity was particularly seen in mRNA-1273 vaccinees. The reasons for the higher within-host diversity among mRNA-1273 vaccinees are not clear. However, several studies have reported higher antibody response and more adverse side effects among mRNA-1273 vaccinees than among BNT162b2 vaccinees.^35^^,^^36^^,^^37^ The higher immune response following mRNA-1273 vaccination could exert immune pressure on B.1.617.2 to change and hence may explain the higher within-host diversity in those patients. Interestingly, the mRNA-1273 vaccine was found to be more effective in preventing B.1.617.2 infection than the BNT162b2 vaccine.^38^^,^^39^^,^^40^ Unlike other lineages, the higher diversity seen among vaccinated Beta individuals was derived from BNT162b2-vaccinated individuals. This could be related to the higher number of samples than mRNA-1273-vaccinated individuals. This was due to the limited use of the Moderna vaccine during the Beta variant outbreak in the country. However, this may suggest that the higher immune response elicited following mRNA-1273 vaccination has resulted in higher within-host mutations and hence a broader immune response which offered better protection against Delta-B.1.617.2 infections. Overall, the mRNA-1273 vaccine has also demonstrated better effectiveness against other variants than BNT162b2. Yet, no significant difference in within-host diversity was found between mRNA-1273- and BNT162b2-vaccinated individuals in all other variants.

In addition to overall diversity, we examined the emergence, frequency, and spread of immune-escape mutations, especially among vaccinated individuals. Current data estimated that 65% of the world population has received at least 1 dose of vaccine.^41^ In a highly seropositive population, the emergence of immune escape mutations is inevitable. Reports on vaccine breakthrough infections in vaccinated individuals are also accumulating, raising concern of escape mutations emergence as a result of immune selection.^42^^,^^43^ The emergence of immune-escape mutations was clearly demonstrated at later stages in long-infected immunocompromised patients.^23^ Here, we reported, despite being rare, the emergence of immune-escape mutations in different variants. Of these mutations, R346K in S-RBD was of particular interest. In our data, this mutation appeared first at low prevalence in Delta-B.1.617.2 and Omicron-B.1.1.529, then at a higher prevalence in BA.1. Later on, BA.1 sequences carrying R346K mutation were assigned to a new Omicron sub-lineage, BA.1.1. However, the first appearance of R346K mutation was reported in the Mu (B.1.621) variant which appeared in early 2021 in South America and spread later on to Europe.^44^ Prediction and experimental methods showed that this mutation can escape recognition by more than 10 monoclonal antibodies.^45^^,^^46^ Another escape mutation of interest is S255F in NTD of S. In our study, this mutation was seen in Delta lineages: B.1.617.2 and AY.4. In B.1.617.2-positive samples, S255F was detected in 10 samples (out of 81) and particularly among vaccinated individuals (>90%). Its prevalence among unvaccinated samples was higher (50%) in AY.4 samples. Unlike R346K, this mutation is not associated with any specific variant of concern. S255F is located within multiple T- and B-cell epitopes and was found to be associated with reduced neutralization by a monoclonal antibody, mAb_S2L28.^27^ Despite its immune-escape properties, this mutation was not fixed in Delta lineages, and its prevalence remained limited. Taken together, putative within-host mutations may emerge in antigenic sites, particularly in vaccinated samples. However, few may rise in frequency and prevalence.

### Conclusion

Since the first identification of SARS-CoV-2, hundreds of variants have emerged and spread globally causing multiple waves of infection. As the virus circulate in seropositive populations, more variants are expected to rise due to the immune pressure of previous infection and/or vaccination. Pre-existing immunity is expected to maximize the number of within-host mutations and result in higher within-host diversity. Here, we reported relatively higher within-host diversity among vaccinated individuals, particularly among Beta- and Delta-B.1.617.2-infected individuals. We have also investigated the emergence of immunity-evading mutations and reported, despite being rare, mutations in Delta and Omicron lineages. Within-host mutations with resistance against natural or vaccine-induced immunity would probably be selected and replace previously circulating strains. Therefore, the continuous tracking of novel and potentially clinically important mutations is of great importance in light of public health, disease control, and the design of new preventive immunization strategies.

### Limitation of the study

This study has some limitations that should be addressed. We could not sequence the effective sample size of some groups (STAR Methods section). We did not study other “variants of concern” as those were not detected in Qatar. It is noteworthy to mention that more than 26 Delta sub-lineages were circulating in Qatar during the period between May and November 2022; however, those were not included in this analysis. Only the most prevalent Delta sub-lineages, B.1.1617.2 and AY.4, were included. Future work should focus on studying changes in within-host diversity in fully vaccinated individuals who received three doses of the vaccine. It should also investigate the impact of other vaccine types (non-RNA-based vaccines) on within-host diversity.

## STAR★Methods

### Key resources table

**Table:** 

REAGENT or RESOURCE	SOURCE	IDENTIFIER
**Critical commercial assays**		

CleanPlex SARS-CoV-2 Research and Surveillance panel	Paragon Genomics	Cat#918002
CleanPlex for MGI Single-Indexed PCR Primers	Paragon Genomics	Cat#318007
CleanMag Magnetic Beads	Paragon Genomics	Cat# 718003
MGIEasy circularization kit	MGI	Cat#1000005259
DNBSEQ-G50RS High-throughput Sequencing Set	MGI	Cat# 1000019859
Qubit RNA HS assay kit	Invitrogen	Cat# Q32852

**Deposited data**		

NCBI BioProject ID PRJNA863945	This paper	https://www.ncbi.nlm.nih.gov/bioproject/?term=PRJNA863945

**Software and algorithms**		

SARS-CoV-2_Multi-PCR_V1.0 pipeline	MGI Tech bioinformatics	https://github.com/MGI-tech-bioinformatics/SARS-CoV-2_Multi-PCR_v1.0
SOAPnuke filtration toolkit (version 2.0.6)	Chen at al 2018^47^	https://github.com/BGI-flexlab/SOAPnuke
BWA (version 0.7.17)	Li 2013^56^	https://github.com/lh3/bwa
Samtools (version 1.7)	Danecek et al. 2021^50^	https://github.com/samtools/samtools
Fgbio software package		https://github.com/fulcrumgenomics/fgbio
LoFreq (version 2)	Wilm et al. 2012^55^	https://csb5.github.io/lofreq/
GraphPad Prism 9.0		www.graphpad.com
R (version 4.2.1)		www.R-project.org

### Resource availability

#### Lead contact

Further information and requests for resources and reagents should be directed to and will be fulfilled by the lead contact, Hebah A. Al-Khatib (h.alkhatib@qu.edu.qa).

#### Materials availability

All sequences generated in this study are publicly available in NCBI website (NCBI BioProject ID PRJNA863945).

### Experimental models and subject details

#### Sample selection criteria

Nasopharyngeal swabs were collected and tested in the virology laboratory at Hamad Medical Corporation, Qatar. Aliquots of viral transport medium of positive samples (Ct value <25) were transported to be sequenced in the Biomedical Research Center at Qatar University. A representative number of samples were selected from the four SARS-CoV-2 pandemic waves: Alpha (December 2020-March 2021), Beta (February 2021-April 2021), Delta (April 2021-November 2021), and Omicron (December 2021-now) (Table 1). To avoid contamination and spillover across variants, samples were selected from the peaks of each wave during which only one lineage was dominating in the country. Samples were selected from patients with no history of SARS-CoV-2 infection. Further, samples were selected from patients aged between 12- and 60-years old with mild to moderate symptoms. These selections were made based on our previous results that displayed higher within-host mutations in elderly (older than 60 years old) and severely ill patients.^22^ Also, only samples collected within 1–3 days following the onset of symptoms were selected to minimize variations in within-host diversity reported over the course of infection as previously reported.^24^ Finally, only samples with Ct values ranging from 18 to 22 were selected. Lythgoe et al. (2021) reported that calling within-host mutations at a minimum frequency of 3% is highly reproducible for samples having 50,000 uniquely mapped reads which correspond to a cycle threshold of ∼22. Selection of samples based on age range, infection severity and Ct values was done to minimize their known effect on intra-host diversity and focus on the impact of lineage, vaccination status and vaccination type. A total number of 379 samples were selected for deep sequencing analysis (Table 1).

Sample size calculation was performed to determine the number of samples required within each group. However, this number could not be achieved for some study groups due to the following issues:

• Vaccinated individuals infected with Alpha variant. None of Alpha positive cases had received SARS-CoV-2 vaccine. During the Alpha peak that lasted during January and March 2021, the use of vaccine to restricted to high-risk groups and hence it was difficult to find vaccinated individuals who fit the above-mentioned criteria.
• Moderna-vaccinated individuals infected with Beta variant. Pfizer vaccine was the main vaccine used during the circulation of Beta variant (February 2021-May 2021). The majority of Moderna vaccinated individuals had received their first dose of vaccine only and hence were not included in the analysis.
• Omicron-B.1.1.529 samples. This Omicron variant circulated for only 2 weeks in Qatar and hence we could not find enough number of samples.

#### Ethical approval

This study was approved by the IRB committees of Qatar Biobank (QF-QBB-RES-ACC-0184).

#### Extraction and quantification of viral RNA

Viral RNA was extracted from 150 uL of viral transport medium of nasopharyngeal and/or oropharyngeal samples using the MGISP-960 sample preparation system (MGI, China). Viral load was quantified using qPCR (RT-qPCR) using TaqPath COVID-19 Combo Kits (Thermo Fisher Scientific, USA) on an ABI 7500 FAST (Thermo Fisher Scientific, USA) that targets the viral S, N, and ORF1ab gene regions. Viral load was then estimated from Ct values against a standard curve that has been generated using a serially diluted viral RNA control (0–200,000 copies/reaction).

#### Sequencing full-length SARS-CoV-2 genome

Libraries were generated using the CleanPlex SARS-CoV-2 Research and Surveillance panel as described by the manufacturer (Paragon Genomics, China). Briefly, extracted RNA was quantified using a Qubit RNA HS assay kit and 100 ng of viral RNA was used for the reverse transcription step. This was followed by a multiplex PCR reaction using two sets of primer pools to ensure full coverage of the viral genome. All primers sequences used to sequence the variants can be requested as bed files from Paragon Genomics company. A second PCR was then performed to add specific indexes to each sample. PCR products were purified using CleanMag Magnetic Beads (Paragon Genomics, China). Indexed libraries were quantified, normalized, and pooled to generate a final yield of 155 ng of DNA. Pooled libraries were then converted into single-stranded DNA and circularized using the MGIEasy circularization kit (MGI, China) as instructed in the protocol. Circularized DNA was bead-purified and used for DNA nanoball (DNB) generation. DNBs were quantified and at least 800 ng were loaded in the MGI-G50 sequencer. Each sequencing run included 94 samples, negative buffer control, and an RNA extracted from non-COVID-19 patients. All sequencing runs were performed using DNBSEQ-G50RS High-throughput Sequencing Set that includes the large, paired-end flow cell (FCL PE100, MGI).

#### Analysis of next-generation sequencing data

Analysis of sequence reads was performed using the SARS-CoV-2_Multi-PCR_V1.0 pipeline available at GitHub (https://github.com/MGI-tech-bioinformatics/SARS-CoV-2_Multi-PCR_v1.0). In short, demultiplexed sequence read pairs were trimmed to remove the adaptors and primer sequences using the SOAPnuke filtration toolkit.^47^ Trimmed reads were then mapped to the SARS-CoV-2 Reference genome Wuhan-Hu-1 (GenBank: NC_045512.2) using bwa mem version 1.5.7 as the mapper, and samtools were used for the final analysis.^48^^,^^49^^,^^50^ Only properly paired reads with insert size <500 bp and with at least 90% sequence identity to the reference were retained. Primer sequences were then masked from mapped reads (BAM files) using fgbio software package.^51^ Clean mapped reads were then used for consensus sequence construction and variant calling. Variants were called using Freebayes variant detector tools and were restricted only to positions with a minimum depth of 100, frequency of 60%, and quality of 30.^52^ For analysis of consensus genomes, consensus calls required a minimum of ten uniquely mapped reads per position. Lineages were assigned by the Pangolin web server using the determined consensus genome for each sequenced sample.^53^^,^^54^

#### Intra host single nucleotide variants (iSNVs) calling

Full coverage sequences (>95% coverage) were considered for subsequent within-host diversity analysis. Previous studies have estimated within-host diversity by evaluating the number of within-host single nucleotide variants (iSNVs) occurring above a specific minor allele frequency (MAF) threshold. Here, we evaluated the within-host diversity by counting the number of single-nucleotide variants (iSNVs) in each sample including (i) mutations occurring above the minor allele frequency (MAF) threshold of 5%; (ii) mutations with a minimum sequencing depth threshold of 500 reads; (iii) mutations not occurring in RNA control, and (iv) mutations occurring in coding regions. MAFs were computed at every position using low-frequency variants calling tools: LoFreq and Freebayes, with the default parameters of no indel calling and a maximum pileup depth of 1,000,000.^55^^,^^56^ The ESC and IEDB (https://www.iedb.org) resources were used to investigate the immune escape properties of within-host mutations.^45^ Positive selection in each site was estimated using the Bayesian approach, FUBAR (Fast, Unconstrained Bayesian Approximation), which infers non-synonymous (dN) and synonymous (dS) substitution rates in each position for a given coding alignment assuming a constant selection pressure for each position for all sequences in the alignment.

#### Statistical analysis

Comparison of within-host mutations between samples of each group and among different groups was determined using the one-way ANOVA followed by a Kruskal–Wallis test (within-group) and post-hoc Dunn’s multiple comparisons test (between groups) using GraphPad Prism 9. Linear model analysis was performed to study the relationship between lineage and vaccine type using the R. The R package emmeans was used to calculate the estimated marginal means, the broom package was used to format the linear model results in a readable manner and ggblot for visualization. The full linear model is described in File S1. Significance was considered for p values < 0.05.

## Data Availability

This paper does not report original code. Data reported in this paper and any additional information required to reanalyze the data will be provided from the lead contact upon request.
