# Supplementary material for: Comparative analysis of within-host diversity among vaccinated COVID-19 patients infected with different SARS-CoV-2 variants
Source: iScience. 2022 Oct 25;25(11):105438. doi: 10.1016/j.isci.2022.105438 (PMC9595287; doi:10.1016/j.isci.2022.105438)
Supplement: Document S1. Figure S1 and File S1 [file mmc1.pdf]

iScience, Volume *25*

Supplemental information

Comparative analysis of within-host diversity among vaccinated COVID-19 patients infected with different SARS-CoV-2 variants

Hebah A. Al-Khatib, Maria K. Smatti, Fatma H. Ali, Hadeel T. Zedan, Swapna Thomas, Muna N. Ahmed, Reham A. El-kahlout, Mashael A. Al Bader, Dina Elgakhlab, Peter V. Coyle, Laith J. Abu-Raddad, Asma A. Al Thani, and Hadi M. Yassine

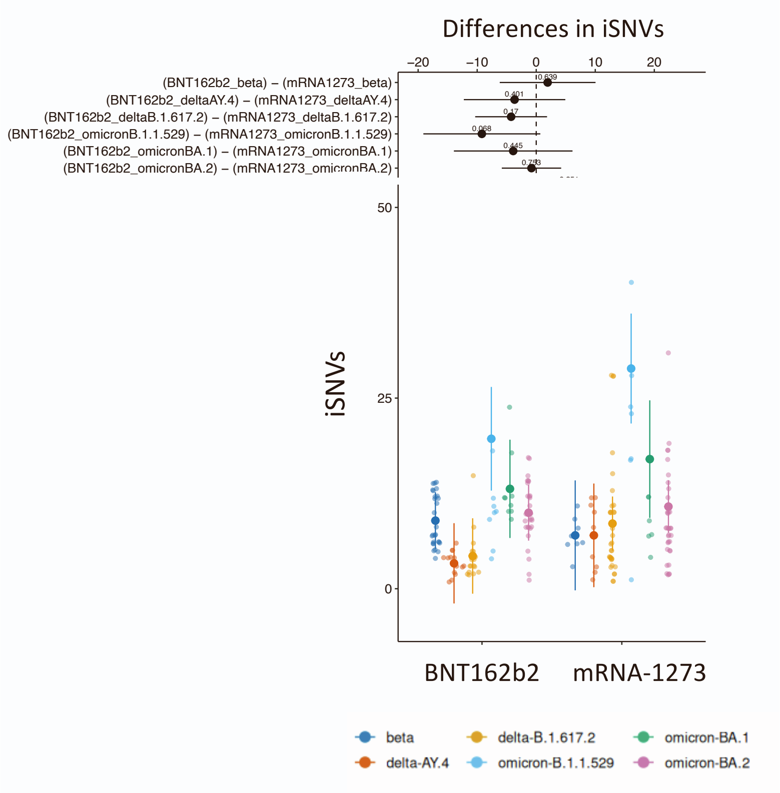

Supp. Figure 1: Linear model displaying the interaction effect of lineage and vaccine type on iSNVs prevalence. The linear model was performed using R packages: emmeans for calculating the estimated marginal means, broom package for format the linear model results in a readable manner and ggblot for visualization. Statistical significance was considered for p-values < 0.05. This figure is related to **Figure 3**. The full linear model is described in **supp. file 1.**

Supp. file 1: Full model analysis of interaction between virus_lineage and vaccine type. This data is related to supp. figure 1 and to the “Statistical analysis” section in Star Methods.

```
Call:
lm(formula = mutations ~ vaccine_type:virus_lineage, data =
InteractionDataset)

Residuals:
    Min      1Q  Median      3Q     Max
-27.875  -4.000  -1.100   2.008  78.333

Coefficients: (1 not defined because of singularities)
                                             Estimate Std. Error t
value Pr(>|t|)
(Intercept)                                   10.7714     1.7474
6.164 3.84e-09 ***
vaccine_typeBNT162b2:virus_lineagebeta                 -1.8381
2.5721  -0.715   0.4757
vaccine_typemRNA-1273:virus_lineagebeta                -3.7714
4.0512  -0.931   0.3530
vaccine_typeBNT162b2:virus_lineagedelta-AY.4           -7.4381
3.1903  -2.331   0.0207 *
vaccine_typemRNA-1273:virus_lineagedelta-AY.4          -3.7714
3.8636  -0.976   0.3302
vaccine_typeBNT162b2:virus_lineagedelta-B.1.617.2      -6.4773
3.0561  -2.119   0.0353 *
vaccine_typemRNA-1273:virus_lineagedelta-B.1.617.2     -2.2260
2.5084  -0.887   0.3759
vaccine_typeBNT162b2:virus_lineageomicron-B.1.1.529     8.8952
3.8636   2.302   0.0223 *
vaccine_typemRNA-1273:virus_lineageomicron-B.1.1.529  18.1036
4.0512   4.469 1.32e-05 ***
vaccine_typeBNT162b2:virus_lineageomicron-BA.1          2.3286
3.7068   0.628   0.5306
vaccine_typemRNA-1273:virus_lineageomicron-BA.1         6.2286
4.2802   1.455   0.1472
vaccine_typeBNT162b2:virus_lineageomicron-BA.2         -0.8037
2.5497  -0.315   0.7529
vaccine_typemRNA-1273:virus_lineageomicron-BA.2             NA
NA      NA       NA
---
Signif. codes:  0 ‘***’ 0.001 ‘**’ 0.01 ‘*’ 0.05 ‘.’ 0.1 ‘ ’ 1

Residual standard error: 10.34 on 200 degrees of freedom
Multiple R-squared:  0.2084,      Adjusted R-squared:  0.1649
F-statistic: 4.787 on 11 and 200 DF,  p-value: 1.572e-06

   term                                        estimate std.error
statistic  p.value ConfidenceInterval(low) ConfidenceInterval(high)
```

```
 1 (Intercept)                                  10.8      1.75   6.16
3.84e-9    7.33  14.2
 2 vaccine_typeBNT162b2:virus_lineagebeta            -1.84     2.57
-0.715  4.76e-1   -6.91   3.23
 3 vaccine_typemRNA-1273:virus_lineagebeta           -3.77     4.05
-0.931  3.53e-1  -11.8    4.22
 4 vaccine_typeBNT162b2:virus_lineagedelta-AY.4      -7.44     3.19
-2.33   2.07e-2  -13.7   -1.15
 5 vaccine_typemRNA-1273:virus_lineagedelta-AY.4     -3.77     3.86
-0.976  3.30e-1  -11.4    3.85
 6 vaccine_typeBNT162b2:virus_lineagedelta-B.1.617…  -6.48     3.06
-2.12   3.53e-2  -12.5   -0.451
 7 vaccine_typemRNA-1273:virus_lineagedelta-B.1.61…  -2.23     2.51
-0.887  3.76e-1   -7.17   2.72
 8 vaccine_typeBNT162b2:virus_lineageomicron-B.1.1…   8.90     3.86
2.30   2.23e-2    1.28  16.5
 9 vaccine_typemRNA-1273:virus_lineageomicron-B.1.…  18.1      4.05
4.47   1.32e-5   10.1   26.1
10 vaccine_typeBNT162b2:virus_lineageomicron-BA.1     2.33     3.71
0.628  5.31e-1   -4.98   9.64
11 vaccine_typemRNA-1273:virus_lineageomicron-BA.1    6.23     4.28
1.46   1.47e-1   -2.21  14.7
12 vaccine_typeBNT162b2:virus_lineageomicron-BA.2    -0.804    2.55
-0.315  7.53e-1   -5.83   4.22
13 vaccine_typemRNA-1273:virus_lineageomicron-BA.2   NA       NA
NA     NA         NA     NA

vaccine_type virus_lineage          emmean   SE  df lower.CL upper.CL
 BNT162b2  beta                   8.93 1.89 200    5.212    12.66
 mRNA-1273 beta                   7.00 3.65 200   -0.207    14.21
 BNT162b2  delta-AY.4             3.33 2.67 200   -1.930     8.60
 mRNA-1273 delta-AY.4             7.00 3.45 200    0.205    13.79
 BNT162b2  delta-B.1.617.2        4.29 2.51 200   -0.650     9.24
 mRNA-1273 delta-B.1.617.2        8.55 1.80 200    4.997    12.09
 BNT162b2  omicron-B.1.1.529     19.67 3.45 200   12.872    26.46
 mRNA-1273 omicron-B.1.1.529     28.88 3.65 200   21.668    36.08
 BNT162b2  omicron-BA.1          13.10 3.27 200    6.654    19.55
 mRNA-1273 omicron-BA.1          17.00 3.91 200    9.295    24.70
 BNT162b2  omicron-BA.2           9.97 1.86 200    6.306    13.63
 mRNA-1273 omicron-BA.2          10.77 1.75 200    7.326    14.22

Confidence level used: 0.95

 contrast                                                  estimate
SE  df t.ratio p.value
 (BNT162b2_beta) - (mRNA1273_beta)                            1.933
4.11 200   0.470  0.6389
 (BNT162b2_deltaAY.4) - (mRNA1273_deltaAY.4)                 -3.667
4.36 200  -0.841  0.4012
```

```
 (BNT162b2_deltaB.1.617.2) - (mRNA1273_deltaB.1.617.2)       -4.251
3.09 200  -1.378  0.1699
 (BNT162b2_omicronB.1.1.529) - (mRNA1273_omicronB.1.1.529)   -9.208
5.02 200  -1.833  0.0683
 (BNT162b2_omicronBA.1) - (mRNA1273_omicronBA.1)             -3.900
5.09 200  -0.766  0.4449
 (BNT162b2_omicronBA.2) - (mRNA1273_omicronBA.2)             -0.804
2.55 200  -0.315  0.7529
```
